# Effects of Seed Endophytic Bacteria on Life History and Reproductive Traits in a Cosmopolitan Weed, *Capsella bursa-pastoris*

**DOI:** 10.3390/plants11192642

**Published:** 2022-10-08

**Authors:** Byungwook Choi, Tae-Min Kim, Seorin Jeong, Yousuk Kim, Eunsuk Kim

**Affiliations:** School of Earth Sciences and Environmental Engineering, Gwangju Institute of Science and Technology, Gwangju 61005, Korea

**Keywords:** plant fitness, life history, reproductive traits, seed bacteria, seed germination, selection

## Abstract

Diverse bacteria inhabit plant seeds, and at least some of them can enhance plant performance at the early developmental stage. However, it is still inconclusive whether seed bacteria can influence post-germination traits and their contribution to plant fitness. To explore the evolutionary and ecological consequences of seed endophytic bacteria, we isolated four bacterial strains from the seeds of an annual weedy plant species, *Capsella bursa-pastoris*, and conducted a common garden experiment using seeds inoculated by isolated bacteria. Seeds infected by bacteria tended to germinate in spring rather than in autumn. Bacterial treatment also altered the expression of plant life history and reproductive traits, including flowering dates, rosette diameter at bolting, number of inflorescences, and fruit production. The results of the path analyses suggested that such effects of bacterial treatments were due to bacterial inoculation as well as germination delayed until spring. Spring germinants with bacterial infection showed a weaker association between post-germination traits and relative fitness than those without bacterial infection. These results suggest that seed bacteria likely affect the expression of post-germination traits directly or indirectly by delaying the germination season. An altered contribution of plant traits to relative fitness implies the influence of seed bacteria on the strength of natural selection.

## 1. Introduction

Recent studies have demonstrated the significance of the bacterial microbiome inside plants on plant performance and its potential to mediate local adaptation [1,2,3]. Among those in diverse plant tissues, bacteria occurring inside seeds have received attention because they can be vertically transmitted from parents to offspring to exert beneficial effects across plant generations [4,5]. Some bacteria isolated from seeds can enhance plant performance during the early developmental stage, such as the seed germination rate and seedling growth [6]. Nevertheless, the evolutionary and ecological consequences of seed endophytic bacteria have only recently been evaluated [7,8].

From an evolutionary perspective, bacterial effects on plant life history and reproductive traits are of particular interest, as they are closely related to plant fitness [9]. Bacteria in the rhizosphere are known to affect plant traits because their plant growth-promoting (PGP) traits can increase nutrient availability and modulate phytohormone balance [10,11]. Seed bacteria also exhibit PGP traits [12,13,14]. They can produce plant hormones such as indole acetic acid (IAA) and enzymes involved in hormone metabolism such as 1-aminocyclopropane-1-carboxylate (ACC) deaminase. In addition, seed bacteria can increase phosphate availability by producing phosphatases and facilitate nutrient absorption by producing siderophores. Similar to the interaction between plants and bacteria in the rhizosphere, seed bacteria are expected to affect the expression of post-germination traits.

Alternatively, but not exclusively, if seed bacteria can modulate seed germination behavior, altered germination time may have cascading effects on the expression of life histories at later developmental stages [10,15]. Seed germination is a critical life history event that determines the environmental conditions in which the plant grows [15]. Altered germination time exposes plants to different environmental cues, affecting the expression of plastic post-germination traits at later developmental stages [16,17]. Seed bacteria may have the ability to affect plant life histories directly by changing plant physiology or indirectly by altering the timing of seed germination.

Altered post-germination traits might contribute to lifetime fecundity differently. According to Cheplick [18], the existence of seed microbes may affect the associations between plant traits and relative fitness because they can influence plant development or environmental conditions in plant habitats. For instance, if seed bacteria can increase nutrient availability, altered nutrient availability would change the contribution of plant traits to plant fitness. The effect of seed bacteria on germination time may be another factor. Changing environmental conditions due to altered germination time can affect the association between post-germination traits and plant fitness [15,19].

To explore the ecological significance of seed bacteria, we isolated bacteria from the seeds of *Capsella bursa-pastoris* and examined whether the inoculation of isolated bacterial strains affected germination time and post-germination traits. *C. bursa-pastoris* is an annual weedy species distributed globally under various conditions, except near the equator [20]. It exhibits a winter or summer annual life cycle, such that seeds germinate in autumn or spring [21,22]. A previous study demonstrated that diverse bacteria inhabited the seeds of *C. bursa-pastoris* and bacterial community structure differed among *C. bursa-pastoris* populations in Korea [14]. Some isolated bacterial strains stimulated shoot growth of seedlings when plants were grown under a controlled growth chamber condition [14].

A common garden was constructed and seeds with and without bacterial inoculation were grown in a field environment. The life history and reproductive traits expressed in later developmental stages were measured. The following questions were addressed: (i) Does seed bacterial inoculation influence germination behavior and post-germination traits in *C. bursa-pastoris*? (ii) Do bacterial inoculation and altered germination season by bacterial treatment influence the expression of post-germination traits? (iii) Do bacterial treatments alter the contribution of plant traits to the lifetime fecundity, a major fitness component of annual plant species?

## 2. Materials and Methods

### 2.1. Seed Collection

**Figure 1:**
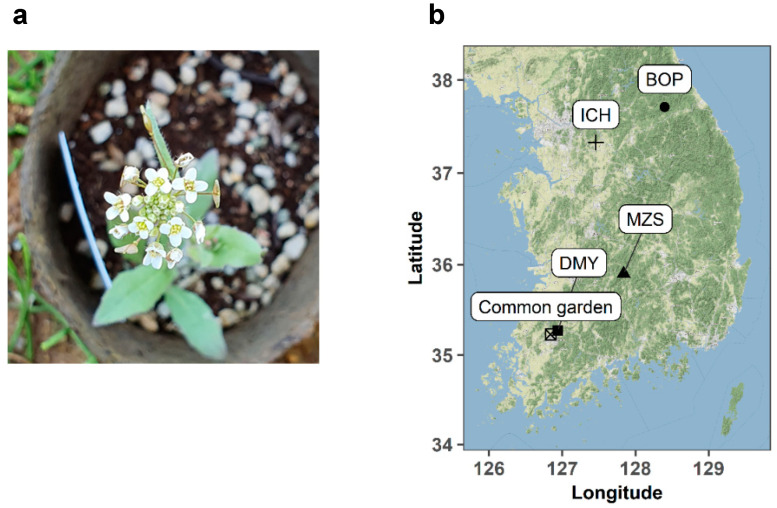
A photograph of *C. bursa-pastoris* (**a**) and locations of seed sources and common garden experiment (**b**). The GPS coordinates and climatic conditions of source populations are given in Table S1. BOP, Bongpyeong, Gwanwon-do; ICH, Icheon, Gyeonggi-do; MZS, Muzu, Jeollabuk-do; DYM, Damyang, Jeollanam-do.

Seeds were collected from more than 20 individuals (maternal genotypes) at four different sites from May to June 2018 (Figure 1, Table S1). Maternal plants were separated with a minimum distance of 5 m and treated as maternal genotypes in this study. Four sites at different latitudes and altitudes were chosen to examine diverse plant genotypes because *C. bursa-pastoris* may exhibit genetic variation along latitudinal and altitudinal gradients [20,23]. Average monthly precipitation was similar across all four sites (Table S1). Thirteen maternal genotypes from each population were randomly chosen to use for the isolation of bacteria from the seeds and to conduct the common garden experiment.

### 2.2. Bacterial Isolation, Identification, and Phenotyping

Seeds from 13 genotypes of each plant population were mixed and endophytic bacteria were isolated and identified as described by Choi et al. [14]. Briefly, surface-sterilized seeds were ground and mixed with ultrapure water. The mixture was spread on LB agar (#7279, Acumedia, Lansing, MI, USA). Single colonies were sub-cultured twice and preserved in 20% glycerol stock solutions at −80 ℃ until required. For bacterial identification, the 16S rRNA gene was amplified with the primer set of 27F (5′-AGAGTTTGATCMTGGCTCAG-3′) and 1492R (5′-TACGGYTACCTTGTTACGACTT-3′) and the PCR products were sequenced (Macrogen Inc., Seoul, Korea). Nucleotide sequences were compared with previously reported sequences of bacterial strains using EZBiocloud (ChunLab Inc., Seoul, Korea). 

Among isolated bacteria, we assessed the PGP traits of *Bacillus* BLA2, *Bacillus* MLA1, *Pantoea* YLA1, and *Pantoea* ILA1 chosen from each of four plant populations (Table 1). We chose those bacterial strains because bacterial species that have the highest rDNA similarities with isolated strains are known to stimulate plant growth in other plant species [24,25,26,27]. For assays, isolated bacterial strains were individually grown in test tubes containing 7 mL LB medium (#7178, Acumedia) at 28 °C in a shaking incubator (210 rpm) for 3 days. Bacterial cells were harvested by centrifugation and rinsed twice with sterilized deionized water (DW). The cells were suspended in DW to an optical density of 1.2 at 600 nm for all assays. We quantified phosphate-solubilizing ability and the production of siderophore, IAA, and ACC deaminase. Nitrogen-fixation ability was not examined, since the amount of fixed nitrogen by seed or leaf endophytic bacteria is not likely critical for host plants [28]. It has been acknowledged that similar biochemical mechanisms influence the phosphate- and potassium-solubilizing ability, so we chose to assess the phosphate-solubilizing ability [29].

Phosphate-solubilizing ability was examined as described by Nautiyal [30]. Briefly, 20 μL of prepared bacterial solution was inoculated in 8 mL of the National Botanical Research Institute’s Phosphate (NBRIP) growth medium (10 g/L glucose, 5 g/L MgCl_2_·6H_2_O, 0.25 g/L MgSO_4_·7H_2_O, 0.2 g/L KCl, 0.1 g/L (NH_4_)_2_SO_4_; pH 6.75) containing 5 g/L Ca_3_(PO_4_)_2_, and incubated at 30 °C in a shaking incubator (150 rpm) for 2 days. The supernatant (100 μL) was mixed with 4.2 mL of sterile DW, 500 μL of 2.5% ammonium molybdate in 5 N sulfuric acid, and 200 μL of α-amino-naphthol solution [31]. After incubation at room temperature for 30 min, the absorbance of the solution was measured at 660 nm using a BioSpectrometer (Eppendorf, Hamburg, Germany). The phosphate concentration was estimated based on a standard curve that ranged from 0.1 to 2 mM phosphate.

Siderophore production was quantified as described by Schwyn et al. [32] and Murakami et al. [33]. We inoculated 20 μL of the prepared bacterial solution into 8 mL of the 10^−2^ diluted LB broth and incubated the broth at 30 °C in a shaking incubator (150 rpm) for 4 days. The supernatant (1 mL) was mixed with 700 μL chrome azurol S (CAS) solution (0.165 g/L CAS, 0.082 g/L FeCl_3_, 0.397 g/L hexadecyltrimethylammonium bromide, in 100 mM piperazine buffer (pH 6.0) with 4 mM 5-sulfosalicylic acid). After incubation for 1 h at room temperature, the absorbance of the solution was measured at 630 nm using a spectrophotometer. The absorbance of the CAS solution with uninoculated media (Ar) and the absorbance of the CAS solution with the supernatant of each sample culture (As) were measured. The percent siderophore unit (psu) was calculated as (Ar − As)/Ar × 100.

IAA was quantified following the method of Patten et al. [34] with some modifications. Prepared bacterial solution (20 μL) was inoculated in 8 mL of LB broth containing 1000 mg/L of L-tryptophan (Sigma-Aldrich) and incubated at 30 °C in a shaking incubator (150 rpm) for 2 days. The supernatant (1 mL) was mixed with 4 mL of Salkowski’s reagent (11.5 M H_2_SO_4_, 9.2 mM FeCl_3_ in sterilized DW). The mixture was incubated for 30 min at room temperature and the absorbance was measured at 535 nm. The IAA concentration was estimated based on a standard curve that ranged from 1 to 50 μg/mL IAA (#I0901.0005, Duchefa, Haarlem, The Netherlands).

The 1-aminocyclopropane-1-carboxylic acid (ACC) deaminase activity was determined following the method of Penrose et al. [35], with some modifications. We inoculated 20 μL of bacterial solution into 7 mL of DF medium (6 g/L Na_2_HPO_4_, 4 g/L KH_2_PO_4_, 2 g/L gluconic acid, 2 g/L citric acid, 0.2 g/L MgSO_4_·7H_2_O, and 1 mL trace element mixtures containing 0.001 g/L FeSO_4_·7H_2_O, 0.01 g/L H_3_BO_3_, 0.011 g/L MnSO_4_·H_2_O, 0.125 g/L ZnSO_4_·7H_2_O, 0.078 g/L CuSO_4_·5H_2_O, and 0.01 g/L MoO_3_) with 3 mM ACC (Sigma-Aldrich, St. Louis, MI, USA) and glucose. The medium was incubated at 30 °C in a shaking incubator (150 rpm) for 4 days. Bacterial cells were collected and rinsed, and the pellet was re-suspended in 600 μL of 0.1 M Tris-HCl (pH 8.5) with 30 μL toluene. To each bacterial mixture (200 μL), 20 μL of 0.5 M ACC was added and incubated at 30 °C for 15 min. After adding 1 mL of 0.56 M HCl, the supernatant was mixed with 800 μL of 0.56 M HCl and 300 μL of 2,4-dinitrophenylhydrazine (0.2% 2,4-dinitrophenylhydrazine in 2 M HCl) (#D2968, Tokyo chemical industry, Tokyo, Japan). After incubation at 30 °C for 30 min, 2 mL of 2 N NaOH was added, and the absorbance at 540 nm was measured using the BioSpectrometer fluorescence. The concentration of α-ketobutyrate was estimated based on a standard curve ranging from 0 to 2 mM α-ketobutyrate (Sigma-Aldrich). The quantity of whole protein was estimated using Lowry’s method [36].

### 2.3. Experimental Design

For bacterial inoculation into seeds, each of four selected bacterial strains was cultured in LB medium at 28 °C in a shaking incubator (180 rpm) until the optical density of the medium reached 1.2 to 1.3 at 600 nm. Then, the bacterial cells were harvested and resuspended in sterile water. Seeds of 13 maternal genotypes from each plant population were surface sterilized using the same procedure used in isolating seed bacteria. Surface-sterilized seeds were immersed in the prepared bacterial suspension or sterile water as a negative control for 24 h at room temperature [37]. The seeds were washed with ultrapure water and stored at room temperature until sowing.

Nine seeds were sprinkled into peat pots (80 mm diameter × 80 mm depth; Jiffy Products of America, Lorain, OH, USA) containing a commercial soil medium for horticultural use (ShinSung Mineral Co., Seongnam-si, Korea). All nine seeds in a pot were from a single maternal plant and inoculated with the same bacterial strain. The soil medium consisted of cocopeat (51.5%), zeolite (10%), perlite (15%), vermiculite (13%), peat moss (10%), and fertilizers (0.4%). The soil manufacturing process includes sterilization of soil components to remove pathogenic microbes.

A common garden was established at Gwangju Institute of Science and Technology on 20 October 2020. A grassy field land (10 m × 7 m) was mowed and tilled to construct the experimental plot. The plot consisted of 6 blocks each of which was 1.1 m wide by 5.7 m long, and access strips 0.5 m wide were made between blocks. Pots were planted into the field ground with a distance of 0.15 m between them. A total of 5 bacterial treatments (4 bacterial isolates and negative control) and 52 maternal genotypes (13 maternal genotypes from each of the 4 plant populations) were randomly positioned within each block following a completely randomized block design. Pots were covered with plastic mesh screens to prevent accidental seed loss.

Seeds germinated from autumn 2020 to spring 2021, except in the winter. To follow the plants systematically, pots with germinants in early November 2020 were labeled as “autumn pots”. “Spring pots” had no autumn germinants but had spring germinants. A focal individual near the center of each pot was chosen in early December 2020 for the autumn pots and in March 2021 for the spring pots. After the focal plant was determined, seedlings other than the focal plant were removed and maintained until the end of the experiment.

Weekly censuses were conducted to record the number of germinants and census to census survivorship of plants from October 2020 to May 2021. For the focal plant, the rosette diameter at the time of inflorescence development (bolting), the day when the plant started flowering (flowering date), and the height and branch number of the central inflorescence were measured. The number of fruits (siliques) was measured to estimate total lifetime fitness. If a plant died before producing siliques, the fruit number was recorded as zero.

### 2.4. Statistical Analyses

All statistical analyses were performed in R 4.0.5 (R Foundation for Statistical Computing, Vienna, Austria). Seed germination rate was calculated as the number of germinants divided by the number of sowed seeds for each pot. Seeds germinated continuously from autumn 2020 to spring 2021. Because rosettes of early germinates could suppress the germination of remaining seeds in the same pot, our measurement might underestimate the seed germination rate. However, if such effect was consistent in all bacterial treatments, our measurement could be utilized to compare germination behavior between seeds with and without bacterial inoculation. A linear mixed model was used to analyze the germination rate. The model included bacterial treatment as a fixed factor, and the block, plant population, genotype nested by plant population, and genotype by treatment interaction were included as random factors. Separate analyses were conducted using a model with bacterial treatment, population, and treatment by population interaction as fixed factors to compare results between the two statistical models. The germination rate was square-root-transformed to meet the normality assumption of the residuals.

A dataset including pots with germinants was constructed to examine the effects of bacterial treatments on the measured traits and fruit production. The binary germination season (0 for autumn pot and 1 for spring pot) was assigned to each pot as an estimate of the germination season. Thus, if a pot had both autumn and spring germinants, its value of binary germination season was “0”. Around 69% of pots had only spring germinants in this experiment (see Results). If the proportion of spring germinants was calculated as the number of spring germinants divided by the number of autumn germinants for each pot, the distribution of the proportion would be highly skewed toward 100% spring germination, which could lower the power of statistical analysis. Thus, although our measurement could overestimate the proportion of spring germination, we used the number of pots instead of the number of germinants to define the germination season.

The tested traits included the binary germination season, binary bolting success (0 for plants that died before bolting or did not bolt until the end of the experiment and 1 for bolting plants), rosette diameter at bolting, flowering date, inflorescence branch number, inflorescence height, and fruit number. Binary germination season and bolting success were analyzed using a generalized mixed model with a binomial error distribution, and the other traits were analyzed using linear mixed models. The model included the same fixed and random factors for the germination rate. Rosette diameter at bolting, inflorescence height, and fruit number were square-root-transformed. Flowering date and inflorescence branch number were natural log-transformed to meet the normality assumption of the residuals. Dunnett’s post hoc multiple comparison tests were conducted to compare trait values between control and bacterial inoculation.

To disentangle the interactions among bacterial inoculation, the binary germination season, and post-germination traits, we conducted path analyses. The path model was constructed with bacterial treatment as an exogenous variable and all other traits as endogenous variables. Because the germination season may affect post-germination traits [15], we hypothesized that both bacterial inoculation and germination season influenced the expression of all post-germination traits. Given correlations between pre-reproductive and reproductive traits in Brassicaceae plants [38,39], it was also hypothesized that the rosette diameter at bolting and flowering date affected the height and branch number of inflorescence, and all those traits, in turn, influenced the number of fruits. Subsets of the full dataset consisting of the negative control and each bacterial treatment were constructed, and path analyses were conducted separately for each sub-dataset. All trait values except the binary germination season were standardized with zero means and one standard deviation following Mitchell [40]. A dummy variable for bacterial treatment was created by assigning the values of 1 to the bacterial inoculation and 0 to the negative control. Path coefficients were estimated using linear mixed models including block, plant population, and genotype nested by plant population as random factors to control for possible differences in unmeasured traits.

We conducted regression analyses to investigate how post-germination traits were associated with the fitness. We adopted the method of phenotypic selection analysis that has been utilized to estimate the relationship between relative fitness and trait values [18,41]. The analyses were performed separately for each bacterial treatment and control. In addition, fall and spring germinants were analyzed separately because the germination season might affect the association between plant traits and plant fitness [15]. Dead plants before bolting were removed from the dataset. Trait values were standardized with zero means and one standard deviation. The relative fitness was estimated as the fruit number divided by the average fruit number in each bacterial treatment [42]. Selection differential (S) was calculated as the covariance between standardized trait values and relative fitness, and selection gradients (β) were partial regression coefficients of standardized trait values against relative fitness. Standard errors of selection differentials were evaluated using simple regression of each trait on relative fitness, and those of selection gradients were assessed using multiple regression of all traits on relative fitness. The block, plant population, and genotype nested by plant population were included in the regression model to control for possible differences in unmeasured traits among plant genotypes.

Analyses of covariance were conducted to determine whether the path coefficient or regression coefficient depended on the bacterial treatments. The model included interactions between traits and bacterial treatments, and significant interactions indicated that path coefficients or regression coefficients varied among bacterial treatments. To determine the slopes that differed, pairwise comparisons based on Tukey’s method were conducted using the emmeans package [43].

## 3. Results

### 3.1. Bacterial Phenotype

*Bacillus* MLA1 produced 635 mg/L soluble phosphate and 21 μg/mL IAA, which were the highest among isolates (Table 1). *Pantoea* YLA1 and *Pantoea* ILA1 produced more than 20% of siderophore units. *Bacillus* BLA2 exhibited relatively lower activity in all measured traits. Three isolates, except *Bacillus* BLA2, exhibited ACC deaminase activity higher than 30 nmol α-ketobutyrate/mg protein • h.

### 3.2. Effects of Bacterial Inoculation on Plant Traits

**Figure 2:**
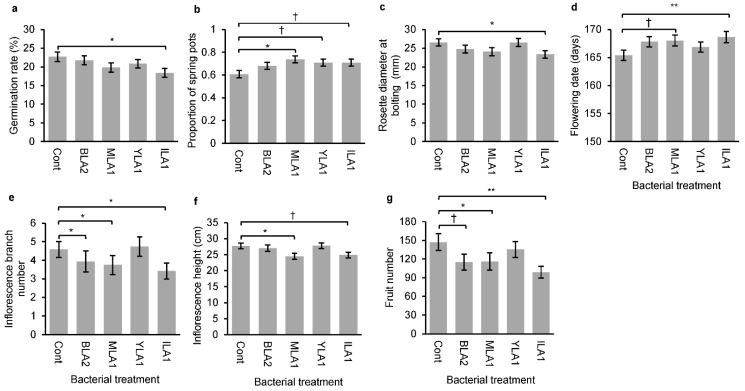
Effects of bacterial treatment on plant traits. Average values and standard errors of (**a**) germination rate, (**b**) proportion of spring germinants, (**c**) rosette diameter at bolting, (**d**) flowering date, (**e**) inflorescence branch number, (**f**) inflorescence height, and (**g**) fruit number are given. Asterisks indicate statistically significant differences between control and each bacterial treatment based on Dunnett’s multiple comparison tests. Abbreviations of bacterial strains are given in Table 1. Cont, control without infection; † *p* < 0.1, * *p* < 0.05, ** *p* < 0.01.

Overall, 72% of pots had at least one germinant, of which 31% contained autumn germinants. The bacterial treatment influenced germination behavior (Table 2). *Pantoea* ILA1 lowered the seed germination rate compared to the control, but the effects of the other bacterial strains were not statistically significant (Table 2, Figure 2a). The binary germination season differed significantly among bacterial treatments (Table 2). Seeds infected by bacteria tended to germinate more in spring than the uninfected seeds, and this treatment effect was observed particularly when *Bacillus* MLA1 was applied to the seeds (Figure 2b).

Bacterial treatment effect was found for most measured post-germination traits (Table 2), and such effects manifested especially in plants inoculated with *Bacillus* MLA1 or *Pantoea* ILA1. Compared to the control plants, plants with *Bacillus* MLA1 or *Pantoea* ILA1 reproduced at smaller rosette size, flowered later, and produced shorter inflorescence with fewer branches and fruits (Figure 2c–g). In contrast, inoculation of *Pantoea* YLA1 did not influence the expression of post-germination traits (Figure 2). Plants infected by *Bacillus* BLA2 exhibited smaller inflorescence branch numbers and fruit production than the control plants (Figure 2e,g).

### 3.3. Direct and Indirect Effects of Bacterial Inoculation on Plant Traits

**Figure 3:**
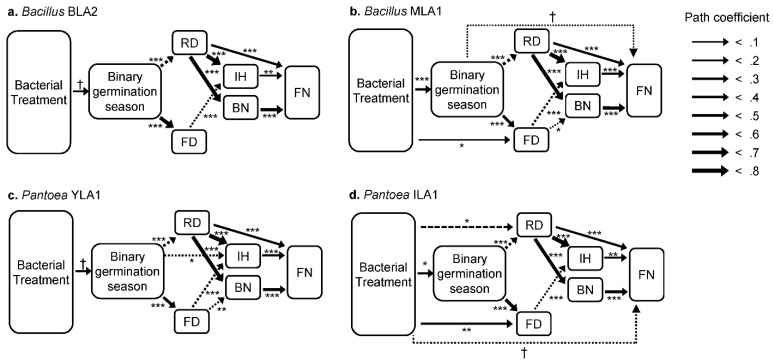
Path analysis models showing the effects of bacterial inoculation on plant life history and reproductive traits. Bacterial treatments are analyzed separately. Solid lines indicate positive path coefficients, and dashed lines indicate negative coefficients. Line thickness is proportional to the value of path coefficient. Only significant paths are shown, and asterisks indicate the statistical significance of path. RD, rosette diameter at bolting; FD, flowering date; IH, inflorescence height; BN, inflorescence branch number; FN, fruit number; † *p* < 0.1, * *p* < 0.05, ** *p* < 0.01, *** *p* < 0.001.

Results of path analyses revealed that the inoculation of *Bacillus* MLA1 and *Pantoea* ILA1 had both direct and indirect effects on the expression of post-germination traits. The *Bacillus* MLA1 and *Pantoea* ILA1 treatment influenced the binary germination season, and the increased spring germination reduced rosette diameter at bolting and delayed flowering date (Figure 3). When the effect of binary germination season was controlled for, bacterial treatment had a direct positive effect on the flowering date in the *Bacillus* MLA1 and *Pantoea* ILA1 treatment, and a direct negative effect on the rosette diameter at bolting in the *Pantoea* ILA1 treatment (Figure 3). The bacterial inoculation in the *Pantoea* ILA1 treatment and the binary germination season in the *Bacillus* MLA1 treatment had a direct negative effect on the fruit number despite the results with marginal significance. In contrast, no direct effect of bacterial treatment or binary germination season was detected for the inflorescence height and branch number.

Analysis of covariance did not detect differences in path coefficients among bacterial treatments (Table S3). However, no direct effect of bacterial treatment on plant traits was observed in the *Bacillus* BLA2 and *Pantoea* YLA1 treatments (Figure 3), indicating that the tested bacterial strains had a differential effect on the expression of post-germination traits.

### 3.4. Association between Post-Germination Traits and Fitness

In the control treatment of fall germinants, regression analyses revealed significant selection differentials for all measured traits (Table 3). Plants exhibited higher relative fitness when they had a larger rosette diameter at bolting, earlier flowering time, and taller inflorescences with more branches. When the inflorescence branch number was controlled for, selection gradients of the other traits were not significant, indicating that their significant selection differentials were probably due to correlation with the inflorescence branch number or unmeasured traits. The results of ANCOVA showed that the contribution of inflorescence branch number to the relative fitness differed among bacterial treatments (Table 3). Compared to the control plants, plants with bacterial inoculation showed larger selection differentials for the branch number when plants were infected with *Bacillus* BLA2 (t = 4.91, *p* < 0.001), *Bacillus* MLA1 (t = 3.02, *p* < 0.05), and *Pantoea* ILA1 (t = 2.85, *p* < 0.05). Larger selection gradients for the branch number were also found in the *Bacillus* BLA2 (t = 4.72, *p* < 0.001), *Bacillus* MLA1 (t = 2.58, *p* = 0.08), and *Pantoea* ILA1 (t = 2.68, *p* = 0.06) treatments. Selection differentials and gradients for other traits were similar across bacterial treatments, as indicated by non-significant trait by treatment interactions (Table 3).

Like the fall germinants, spring germinants also exhibited significant selection differentials for all measured traits in the control treatment (Table 4). However, selection differentials for all traits except flowering date differed among bacterial treatments, as indicated by a significant trait by treatment interaction (Table 4). The *Pantoea* ILA1 treatment showed a lower selection differential than the control for the rosette diameter at bolting (t = 4.42, *p* < 0.001), inflorescence height (t = 3.79, *p* < 0.01), and inflorescence branch number (t = 5.20, *p* < 0.001). A lower selection differential for the rosette diameter was also found in the *Bacillus* BLA2 (t = 2.79, *p* < 0.05) and *Bacillus* MLA1 (t = 3.21, *p* < 0.05) treatments. Selection gradients for the branch number did not differ between control and bacterial treatments even though significant trait by treatment interaction was found (Table 4).

## 4. Discussion

Seeds inoculated with seed bacteria had a higher tendency to germinate in spring than in autumn. Delayed germination, as well as bacterial inoculation, affected the expression of post-germination traits, inducing later flowering time, fewer inflorescences with shorter height, and less fruit production. The association between plant traits and relative fitness differed among bacterial treatments.

### 4.1. Effects of Bacterial Treatments on Seed Germination Behavior

To evaluate the effect of seed bacteria on germination, previous studies have used in vitro experimental settings, such as plate tests, and assessed short-term germination behavior [44]. In contrast, our experiment was conducted under field conditions and demonstrated that the tested bacterial strains could change the binary germination season, i.e., the proportion of pots having only spring germinants. Despite the effect on the germination season, the bacterial treatment rarely influenced the germination rate measured during the experimental period (Figure 2), suggesting that bacterial strains likely influence seed dormancy rather than seed viability.

Germination time is a complex life history trait determined by diverse factors. Both genetic and environmental factors influence the strength and breaking of dormancy [45]. The maternal environment in which the seeds mature is another factor [46]. In combination with these factors, our results suggest that bacteria occurring inside seeds is an additional factor modulating seed germination.

It is still unclear how seed bacteria influence germination behavior [6]. Similar to bacteria in the rhizosphere, seed bacteria have been thought to supply or degrade phytohormones, which might interrupt signaling pathways, and thereby phenological responses of host plants [10,47]. Ethylene is a phytohormone that stimulates seed germination by interacting with gibberellins and abscisic acid [48,49]. Notably, ACC deaminase produced by seed bacteria can degrade ACC, the immediate precursor of ethylene, so it can lower ethylene levels in plant tissues [50]. In this study, all strains except *Bacillus* BLA2 produced ACC deaminase, which may result in delayed seed germination. IAA is another phytohormone that induces seed dormancy and inhibits germination [51]. *Bacillus* MLA1 produced IAA more than the other endophytes (Table 1), and its infection in seeds significantly increased the proportion of spring germination (Figure 2b). If the tested bacterial strains produced IAA and ACC deaminase when seeds were imbibed, this could possibly affect the signaling pathway and germination behavior.

A caveat of this study is that our measurements of germination rate and germination season might not be accurate estimates of germination behavior. We counted the number of germinants without removing early germinants to calculate the germination rate. Because seed germination might be constrained by rosettes of early germinants in the same pot, our measurement might underestimate the seed germination rate. The binary germination season could also underestimate the proportion of spring germination because zero was assigned to autumn pots that might have spring germinants. However, it should be noted that our major purpose was to compare the germination behavior among bacterial treatments. Because the same calculation method is applied to all bacterial treatments, our estimate is likely a reasonable proxy to examine the effect of bacterial inoculation on seed germination behavior.

### 4.2. Effects of Bacterial Treatments on Post-Germination Traits

Treatment effects of bacterial inoculation were detected for most of the measured traits (Table 2). Path analyses revealed that such treatment effects were due to bacterial infection as well as altered germination season caused by bacterial inoculation (Figure 3). It is hypothesized that the impact of seed bacteria on plant performance would manifest at the early developmental stage, and the impact would diminish in plants at later developmental stages [6,12,13]. Our results showed a direct effect of bacterial inoculation on the germination season as well as the size at bolting and flowering time (Figure 3), which are critical life histories affecting plant fitness [38,39]. In addition, seed bacteria indirectly influenced reproductive traits at late developmental stages by altering germination behavior or rosette diameter at bolting (Figure 3). When considering such indirect effects, seed bacteria seem to affect plant performance more than previously postulated.

The shift in the germination season from autumn to spring decreased the rosette diameter at bolting and delayed the flowering date, consequently reducing reproductive trait values including inflorescence height, inflorescence branch number, and fruit production. This is consistent with previous observations that plants with early germination tend to reproduce at a larger size and exhibit higher fecundity due to prolonged growth time [52,53]. Although previous studies have stressed the beneficial effects of seed bacteria [7,8,44], the tested strains decreased plant fecundity, a major fitness component of annual plant species.

However, it should be noted that the fitness consequence of germination behavior highly depends on ecological context. For instance, later germinants may have an advantage in survival if mortality factors occur between the early and late germination times [15]. All the populations examined were from agricultural field sites. In Korea, plowing the ground or applying herbicides in very early spring is a common agricultural practice to remove weeds, such as *C. bursa-pastoris*. In habitats with these mortality factors, spring germination is likely to have a survival advantage over autumn germination. Although delayed germination by seed bacteria would diminish fecundity, it might provide a fitness advantage to plants in habitats with high mortality in winter or early spring.

In this study, the contribution of life history and reproductive traits to the relative fitness differed among the bacterial treatments. Similar results were reported when a fungal endophyte, *Epichloë festucae*, infected the seeds of *Lolium perenne*, such that regression coefficients of morphological traits against relative fitness depended on the infection of *E. festucae* [18]. In addition to fungal species inside the seeds, our results showed that seed bacteria can also alter the association between plant traits and fitness.

Notably, the regression coefficient of a plant trait on relative fitness has been interpreted as the strength of natural or artificial selection [41,42]. Thus, seed bacteria likely act as a selection agent to alter the strength of selection imposed by experimental environmental conditions. In this study, plants were grown in semi-natural environments where plants occurring near focal *C. bursa-pastoris* were regularly removed, but *C. bursa-pastoris* were exposed to ambient temperature, precipitation, and insect herbivores. Given that *C. bursa-pastoris* occurs mainly in highly disturbed habitats similar to our experimental scheme [20,21], our results imply that seed bacteria have the potential to change the intensity of natural selection on plant traits.

Both bacterial infection and altered germination season by bacterial infection can influence the relationship between plant traits and relative fitness [10,15]. When the contribution of plant traits to the relative fitness was assessed for autumn and spring germinants separately, differential contributions of plant traits to the relative fitness were found among bacterial treatments (Table 3 and Table 4). In addition, the *Bacillus* BLA2 treatment altered the selection differential and gradient even though it hardly influenced the germination season (Table 3 and Table 4). These results suggest that bacterial effect, rather than altered germination time, is likely a significant factor affecting the association between traits and relative fitness.

In conclusion, bacterial strains isolated from *C. bursa-pastoris* seeds influenced the germination season as well as post-germination traits, although their impact has been postulated to be manifested in plants at the seedling stage. In addition, seed bacteria can modulate the contribution of plant traits to relative fitness, implying they possibly affect the strength of natural selection.

## Figures and Tables

**Table 1:** Bacterial isolates used in this study and results of plant growth-promoting (PGP) trait assays. GenBank accession number of isolates are shown in the parenthesis and type strains with the highest 16S rDNA sequence similarity are given. Abbreviations of plant populations are shown in Figure 1: -, not detected.

Source Population	Strains (Accession Number)	The Closest Type Strains	rDNA Sequence Similarity (%)	Plant Growth-Promoting Activity (mean ± se)			
				ACC Deaminase (nmol α-KB/mg Protein • h)	Phosphate Solubilization (mg/L of PO_4_)	Siderophore Production (psu, % of Siderophore Units)	Auxin Production (μg/mL)
BOP	BLA2 (ON329310)	*Bacillus altitudinis* 41KF2b	1410/1414 (99.72)	-	27.75 ± 1.36	2.96 ± 1.19	2.68 ± 0.19
MZS	MLA1 (ON329312)	*Bacillus aryabhattai* B8W22	1409/1410 (99.93)	37.56 ± 0.38	653.95 ± 4.93	2.77 ± 1.18	21.16 ± 0.53
DMY	YLA1 (ON329311)	*Pantoea eucalypti* LMG 24198	1339/1347 (99.41)	33.88 ± 0.84	293.24 ± 5.48	24.51 ± 0.28	1.94 ± 0.14
ICH	ILA1 (ON329314)	*Pantoea vagans* LMG 24199	1377/1379 (99.85)	30.51 ± 0.33	87.34 ± 3.60	26.37 ± 0.50	3.87 ± 0.36

**Table 2:** Results of generalized mixed model analyses comparing plant traits among bacterial treatments. The model included block, plant population, genotype nested by plant population, and genotype by treatment interaction as random factors. Chi-square values are given for the binary germination season and bolting success, and F ratios for the other traits are given: * *p* < 0.05, ** *p* < 0.01. Results of analyses using the model with bacterial treatment, population, and population by treatment interaction as fixed factors are given in Table S2. Significant treatment effects were detected for all traits (Table S2).

Trait	Treatment (d.f = 4)
Germination rate	2.74 *
Germination season	10.20 *
Bolting success	0.52
Flowering date	2.75 *
Rosette diameter	2.45 *
Inflorescence height	3.11 *
Branch number	3.50 **
Fruit number	3.64 *

**Table 3:** Results of selection analysis in each bacterial treatment for fall germinants. Results of analyses of covariance are also given to examine differences in the strength of selection among bacterial treatments. Only bolting plants were used. Selection differentials (*S*) and selection gradients (β) with standard errors are shown. *F* ratios for analysis of covariance are given; * *p* < 0.05, ** *p* < 0.01, *** *p* < 0.001. FD, flowering date; RD, rosette diameter at bolting; IH, inflorescence height; BN, inflorescence branch number.

	Control (*N* = 68)		*Bacillus* BLA2 (*N* = 49)		*Bacillus* MLA1 (*N* = 39)		*Pantoea* YLA1 (*N* = 53)	
Traits	*S*	β	*S*	β	*S*	β	*S*	β
FD	−0.43 *** (0.12)	−0.16 (0.10)	−0.07 (0.13)	−0.02 (0.05)	−0.47 ** (0.15)	−0.06 (0.07)	−0.22 (0.11)	−0.02 (0.07)
RD	0.66 *** (0.09)	0.16 (0.16)	0.78 *** (0.11)	0.20 * (0.08)	0.91 *** (0.10)	0.25 * (0.10)	0.78 *** (0.09)	0.37 * (0.15)
IH	0.72 *** (0.09)	0.28 (0.17)	0.49 ** (0.14)	0.12 (0.07)	0.80 *** (0.12)	0.19 * (0.09)	0.73 *** (0.08)	0.27 * (0.13)
BN	0.60 *** (0.09)	0.31 ** (0.11)	1.20 *** (0.07)	0.97 *** (0.07)	0.99 *** (0.07)	0.66 *** (0.08)	0.63 *** (0.10)	0.27 ** (0.10)
	***Pantoea*** **ILA1 (*N* = 44)**		***F_S_*** **(Trait × Treatment)**		***F*** **_β_** **(Trait × Treatment)**			
**Traits**	***S***	**β**						
FD	−0.20 (0.13)	0.05 (0.07)	1.55		1.34			
RD	0.90 *** (0.13)	0.33 ** (0.11)	1.17		0.31			
IH	0.67 *** (0.11)	0.18 (0.11)	0.09		0.56			
BN	0.87 *** (0.08)	0.69 *** (0.07)	8.35 ***		7.72 ***			

**Table 4:** Results of selection analysis in each bacterial treatment for spring germinants. Results of analyses of covariance are also given to examine differences in the strength of selection among bacterial treatments. Only bolting plants were used. Selection differentials (*S*) and selection gradients (β) with standard errors are shown. *F* ratios for analysis of covariance are given; * *p* < 0.05, ** *p* < 0.01, *** *p* < 0.001. FD, flowering date; RD, rosette diameter at bolting; IH, inflorescence height; BN, inflorescence branch number.

	Control (*N* = 96)		*Bacillus* BLA2 (*N* = 111)		*Bacillus* MLA1 (*N* = 115)		*Pantoea* YLA1 (*N* = 109)	
Traits	*S*	β	*S*	β	*S*	β	*S*	β
FD	−0.41 *** (0.12)	−0.04 (0.07)	−0.21 * (0.10)	0.06 (0.05)	−0.23 ** (0.08)	0.01 (0.04)	−0.23 * (0.12)	−0.01 (0.04)
RD	0.98 *** (0.08)	0.32 * (0.13)	0.71 *** (0.07)	0.23 ** (0.07)	0.65 *** (0.05)	0.14 * (0.06)	1.05 *** (0.07)	0.35 *** (0.08)
IH	0.87 *** (0.09)	0.22 (0.11)	0.72 *** (0.07)	0.28 *** (0.07)	0.71 *** (0.05)	0.34 *** (0.07)	0.95 *** (0.08)	0.15 * (0.07)
BN	0.99 *** (0.07)	0.61 *** (0.09)	0.81 *** (0.06)	0.56 *** (0.06)	0.69 *** (0.05)	0.39 *** (0.05)	1.14 *** (0.05)	0.82 *** (0.06)
	***Pantoea*** **ILA1 (*N* = 105)**		***F_S_*** **(Trait × Treatment)**		***F*** **_β_** **(Trait × Treatment)**			
**Traits**	***S***	**β**						
FD	−0.21 ** (0.08)	−0.06 (0.05)	1.03		1.40			
RD	0.52 *** (0.06)	0.29 ** (0.09)	10.03 ***		0.80			
IH	0.48 *** (0.06)	0.03 (0.09)	5.81 ***		1.68			
BN	0.55 *** (0.06)	0.40 *** (0.06)	16.62 ***		7.68 ***			

## Data Availability

All data used in this manuscript are present in the manuscript.

## Supplementary Materials

The following supporting information can be downloaded at: https://www.mdpi.com/article/10.3390/plants11192642/s1, Table S1: Geographic and climatic information of source populations; Table S2: Results of generalized mixed model analyses comparing plant traits among bacterial treatments and plant populations. Table S3: Results of analyses of covariance to compare path coefficients among bacterial treatments.
