# Supplementary material for: Effects of Seed Endophytic Bacteria on Life History and Reproductive Traits in a Cosmopolitan Weed, Capsella bursa-pastoris
Source: Plants (Basel). 2022 Oct 8;11(19):2642. doi: 10.3390/plants11192642 (PMC9570735; doi:10.3390/plants11192642)
Supplement: Supplementary file 1 [file plants-11-02642-s001.zip › plants-1918804-supplementary.pdf]

Table S1. Geographic and climatic information of source populations. Average values for 20 years from 1997 to 2017 are given. MT, monthly mean temperature; MT_S, mean temperature in spring season; MT_A, mean temperature in autumn season; SMP, the sum of monthly precipitation. All values were calculated using the Automatic Weather Station (AWS) database provided by Korea Meteorological Administration (KMA).

Populations	Altitude (m)	Geographic coordinates	MT (°C)	MT_S (°C)	MT_A (°C)	SMP (mm)
BOP	719	37.42'45.7"N 128.24'10.0"E	8.90	7.96	9.47	116.70
MZS	862	35.54'00.8"N 127.50'10.3"E	9.95	9.64	11.27	112.46
ICH	68	37.19'48.0"N 127.27'51.3"E	14.15	13.35	16.09	119.20
DMY	30	35.16'12.5"N 126.56'29.0"E	13.29	12.90	14.84	115.57

Table S2. Results of generalized mixed model analyses comparing plant traits among bacterial treatments and plant populations. The block, genotype nested by plant population, and genotype by treatment interaction were included as random factors. Chi-square values are given for the binary germination season and bolting success, and F ratios for the other traits are given. † *P* < 0.10, * *P* < 0.05, ** *P* < 0.01.

Trait	Treatment (d.f = 4)	Population (d.f = 3)	Treatment × population (d.f = 12)
Germination rate	2.74*	5.68**	1.11
Germination season	9.58*	10.17*	9.75
Bolting success	0.42	14.14**	7.06
Flowering date	2.69*	4.04*	1.24
Rosette diameter	2.22†	2.91*	1.22
Inflorescence height	2.99*	2.38†	2.23*
Branch number	3.43*	2.01	0.97
Fruits number	3.18*	1.88	0.85

Table S3. Results of analyses of covariance to compare path coefficients among bacterial treatments. Significant trait by bacterial strain interaction indicates that path coefficients differed among bacterial strains. * $P < 0.05$.

Path		$F_{trait \times bacterial\ strain}$ (d.f. = 3)
From	To	
Bacterial treatment	Binary germination season	0.44
Bacterial treatment	Rosette diameter at bolting	1.01
Binary germination season		0.88
Bacterial treatment	Flowering date	0.97
Binary germination season		0.66
Bacterial treatment	Inflorescence height	0.37
Binary germination season		0.07
Rosette diameter at bolting		0.07
Flowering date		1.69
Bacterial treatment	Inflorescence branch number	1.34
Binary germination season		0.48
Rosette diameter at bolting		1.50
Flowering date		0.48
Bacterial treatment	Fruit number	0.99
Binary germination season		0.71
Rosette diameter at bolting		0.33
Flowering date		0.18
Inflorescence height		3.47*
Inflorescence branch number		0.78
